# Telephone-Delivered Dietary Intervention in Patients with Age-Related Macular Degeneration: 3-Month Post-Intervention Findings of a Randomised Controlled Trial

**DOI:** 10.3390/nu12103083

**Published:** 2020-10-10

**Authors:** Diana Tang, Paul Mitchell, Gerald Liew, George Burlutsky, Victoria M. Flood, Bamini Gopinath

**Affiliations:** 1 Centre for Vision Research, Department of Ophthalmology and Westmead Institute for Medical Research, University of Sydney, Camperdown, NSW 2006, Australia; paul.mitchell@sydney.edu.au (P.M.); gerald.liew@sydney.edu.au (G.L.); george.burlutsky@sydney.edu.au (G.B.); bamini.gopinath@sydney.edu.au (B.G.); 2 Sydney School of Health Sciences, Faculty of Medicine and Health, The University of Sydney, Camperdown, NSW 2006, Australia; vicki.flood@sydney.edu.au; 3 Western Sydney Local Health District, Westmead Hospital, Westmead, NSW 2145, Australia

**Keywords:** age-related macular degeneration, education, nutrition, telehealth

## Abstract

There is an evidence–practice gap between the dietary recommendations for age-related macular degeneration (AMD) presented in the literature and those practiced by patients. This study reports on the 3-month post-intervention results of a randomised controlled trial (RCT) evaluating telephone-delivered counselling to improve dietary behaviours among AMD patients. A total of 155 AMD patients (57% female, aged 78 ± 8 years; control: 78, intervention: 77), primarily residing in New South Wales, Australia, were recruited. Participants completed a baseline questionnaire and a short dietary questionnaire (SDQ-AMD). The intervention included an evidence-based nutrition resource and four monthly calls with a dietitian. Immediately post-intervention, intervention participants repeated the SDQ-AMD and completed a feedback form. At 3 months post-intervention, both study arms repeated the SDQ-AMD. Statistical analyses included *t*-tests and McNemar’s test. Intervention participants reported satisfaction with the tailored phone calls, nutrition resource and nutrition education provided. At 3 months post-intervention, there was no statistically significant difference between study arms in the proportion of participants meeting the dietary goals nor in intake (mean servings ± SE) of total vegetables (primary outcome) and other key food groups; however, there was a significantly higher intake of nuts (secondary outcome) (3.96 ± 0.51 vs. 2.71 ± 0.32; *p* = 0.04) among participants in the intervention versus control group. Within the intervention arm, there were also significant improvements in intakes of the following secondary outcomes: dark green leafy vegetables (0.99 ± 0.17 vs. 1.71 ± 0.22; *p* = 0.003) and legumes (0.69 ± 0.10 vs. 1.12 ± 0.16; *p* = 0.02) and intake of sweets and processed/prepared foods (8.31 ± 0.76 vs. 6.54 ± 0.58, *p* = 0.01). In summary, although there were few dietary differences between study arms at 3 months post-intervention, the intervention involving four monthly calls was acceptable and helpful to the participants. This type of intervention therefore has the potential to provide people with AMD the needed support for improving their nutrition knowledge and dietary practices, especially if continued over a longer period.

## 1. Introduction

The global prevalence of age-related macular degeneration (AMD) is expected to reach approximately 300 million by the year 2040 [1]. This chronic degenerative disease impairs central vision which is vital for everyday tasks such as reading and driving [1]. The functional disability associated with AMD increases the risk of mental health concerns such as depression and anxiety [2]. Despite the enormous personal burden, treatment options for AMD are limited to costly anti-vascular endothelial growth factor injections for people with the neovascular (wet) form of AMD, while the other form of late AMD (dry atrophic) is irreversible and untreatable [1]. Fortunately, recent research literature supports dietary modulation as a potentially effective preventative and management strategy for AMD [3,4,5]. People with AMD are therefore recommended to increase their consumption of dark green leafy vegetables, fish and low glycaemic index (GI) foods such as wholegrains and legumes [3,4,5,6,7,8].

Despite this, an evidence–practice gap has been identified between these dietary recommendations and those actually practiced by people with AMD [6,9,10,11,12,13,14,15,16,17,18]. This is largely due to a lack of clear-cut guidelines for patients and/or practitioners to follow; patients not having sufficient information and/or misconceptions regarding diet and AMD; inadequate explanation and reinforcement by eye care practitioners; and a lack of dietitian referral and support [6,9,10,11,12,13,14,15,16,17,19,20].

There have been few translational efforts to address this evidence–practice gap. Promising findings were recently published following a non-randomised educational intervention trial in the United Kingdom [21]. In this study, intervention participants received verbal nutrition advice for their AMD from an eye care professional accompanied by a take-home leaflet summarising the key messages around nutrition and AMD; control participants received usual care (off-the-shelf brochure) [21]. As a result of this intervention, participants in this arm increased their egg intake, gained greater confidence about diet and AMD links and were more motivated to practice health-protective behaviours compared to participants in the control arm [21].

Our study is a novel, parallel randomised controlled trial (RCT) that was conducted to assess the effectiveness of a telephone-delivered intervention to impart and disseminate evidence-based dietary advice for people with AMD [22]. A telehealth approach was considered to be appropriate for people with AMD, due to a strong association between older age AMD and its subsequent impact on mobility as a result of central vision loss [1]. Furthermore, telephone-delivered interventions are proven to have several advantages over traditional face-to-face models [23] and were previously shown to improve adherence to dietary recommendations [24,25], diet quality [26] and self-efficacy [24] in both older adults and adults with chronic diseases. Therefore, the aims of this study are to:

1. present the 3-month post-intervention follow-up results of the RCT;
2. report on the feasibility and acceptability of this telehealth programme for AMD patients.

## 2. Materials and Methods

### 2.1. Baseline Period

A total of 155 participants were recruited by study personnel between June 2018 to July 2019. This number exceeds the proposed 140 participants needed to sufficiently achieve the primary outcome with 80% power as significant at the 5% level and allowing for a 10% drop-out rate. Most participants were recruited within New South Wales (*n* = 150, 96.7%), of which 137 participants were recruited from three Sydney-based private eye clinics and 12 participants were recruited from our research database listing people who consented to be contacted about future studies by our research team. The remaining five participants were recruited across Australia following media advertisements.

Inclusion criteria for the study were (a) a physician diagnosis of any form of AMD in either eye and (b) age > 50 years old. These criteria were confirmed by the physician and/or clinical staff at the respective eye clinics and further details about the type of AMD (early or late) were collected from the treating physicians’ notes, that is, study personnel were not involved in the AMD grading process. The following were exclusion criteria: (a) lack of sufficient English fluency; (b) unwillingness to participate in the 4-month intervention programme; and (c) inability to provide informed consent. Participants were successfully recruited when written consent and the following baseline questionnaires were completed and returned: general baseline questionnaire including demographics, medical history and vision function; a 145-item food frequency questionnaire including information on supplement use to capture usual intake over 12 months; and a short dietary questionnaire (SDQ-AMD) [27] to capture actual intake of key food groups in the last seven days. Following recruitment, participants were randomised into one of two arms: intervention or control. The randomisation sequence was generated centrally using permuted blocks of mixed size to ensure a 1:1 allocation ratio while maintaining an unpredictable sequence. Assignments to the intervention or control arm were managed centrally so treating and recruiting staff were not involved in the process, however, neither participants nor research staff could be blinded to the assignment allocations. All participants continued to receive usual care for their AMD by their eye care professional.

This study was conducted in accordance with the Declaration of Helsinki. The protocol for the RCT has been published previously [22] and was approved by the University of Sydney Human Ethics Committee (Reference: HREC 2018/219). The CONSORT checklist (Supplementary File S1) and TIDiER checklist (Supplementary File S2) were completed.

### 2.2. Intervention Period

The intervention ran between August 2018 to October 2019 and included a two-pronged approach: (1) develop and distribute a resource or workbook incorporating messages based on the latest Australian Dietary Guidelines [28] and evidence-based information on diet and AMD to the participant; and (2) have an accredited practising dietitian provide monthly telephone coaching and support for four months to facilitate and enhance the participants’ adoption of AMD-specific dietary recommendations. The key dietary messages of this intervention included: (i) increase consumption of dark green leafy vegetables, (ii) eat fresh fruit daily, (iii) choose low GI foods, (iv) eat fish at least twice per week, and (v) consume nuts two to three times per week.

The anticipated duration of each phone call was 20 min or a total of 80 min over four months. Any missed consultations for the month were logged by the dietitian to assess adherence at the end of the intervention. The number of call attempts and call duration were also logged. The intervention calls were tailored according to the “four As approach” [29]: (1) assessment (feedback) of participant diet and stage of change; (2) advice on optimal dietary behaviours; (3) assistance with collaborative goal setting; and (4) arranging follow-up support, i.e., next phone call [22]. The stages of change used in this study were categorised as: pre-contemplation (not thinking about change); contemplation (thinking about change within the next six months); preparation (making a change within the next month); action (currently trying to change); or maintenance (maintaining a change) [30].

The control arm received freely available off-the-shelf brochures about AMD and nutrition and were also briefly followed up by a member of the research team once per month during the intervention period. Phone call discussions included queries about the control package or study and/or any relevant comments and updates

### 2.3. Follow-Up Period

Immediately following the 4-month intervention period, intervention participants were asked to complete a feedback form and repeat the SDQ-AMD that was administered at baseline. The feedback form was used to determine the acceptability of the intervention and included a 5-point Likert scale to assess satisfaction with the telehealth component and evidence-based resource and two “yes/no” questions: (1) “Would you feel confident in recommending this treatment to a friend?” and (2) “Was it worth your time doing the programme?”. A 5-point Likert scale was also used to determine self-assessed adherence to the intervention programme, and two free-response questions allowed participants to reflect on the most useful component of the intervention and provide additional feedback such as suggestions to improve the programme. A second follow-up was conducted 3 months post-intervention, inviting both intervention and control participants to repeat the SDQ-AMD.

### 2.4. Outcome Measures

As specified in the published protocol [22], the primary outcome measure for this study was a 0.5 serving per day increase in total vegetable intake; an achievable improvement according to data from an Australian population-based intervention [31]. Appreciable improvements in the dietary intakes of dark green leafy vegetables, fruit, low GI foods, fish and nuts were the secondary outcomes in the 3-month post-intervention follow-up period.

### 2.5. Analysis

Questionnaire data were entered into developed templates in REDCap (a secure web-based application for managing online databases). Data were exported as Excel spreadsheets for per-protocol statistical analysis using SAS version 9.4. Chi-squared tests and Fisher’s exact test were used to compare baseline study characteristics between the control and intervention arms and confirm that participants were randomly allocated. Descriptive statistics were used to describe the participants, call frequency, call duration and dietary intakes. *T*-tests were used to compare dietary changes within and between each study arm at applicable follow-up periods with adjustments for age and sex. McNemar’s test was used to compare the change in proportion of participants meeting the dietary goals. Level of significance for all statistical analyses was *p* < 0.05.

## 3. Results

### 3.1. Participants

**Figure 1:**
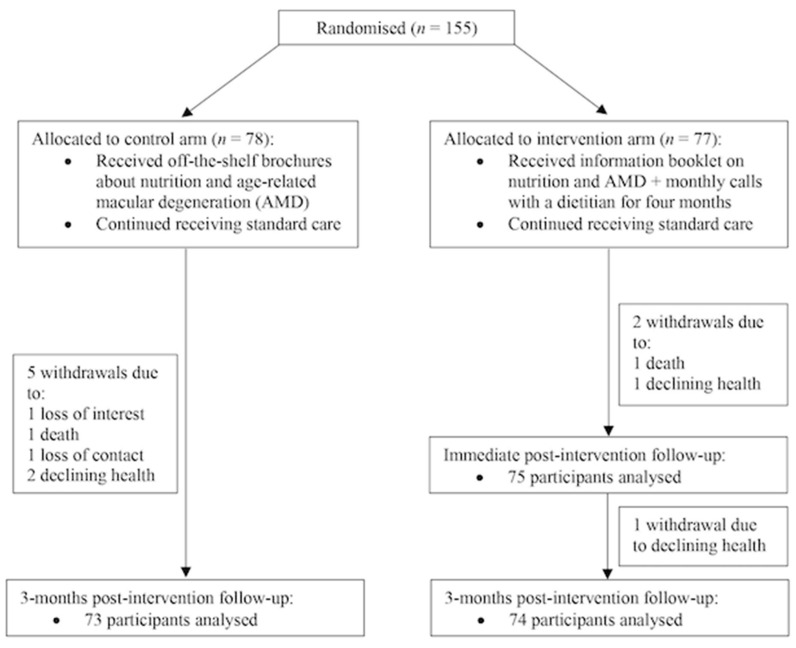
Trial profile.

Figure 1 describes the trial profile where 155 participants were recruited at baseline (control, *n* = 78; intervention, *n* = 77). The overall withdrawal rate at the 3-month post-intervention follow-up was 5% (*n* = 8; three intervention, five control). Participant baseline characteristics are shown in Table 1 and differences in study characteristics between the study arms were mostly non-significant (exceptions for height and type of AMD).

Within the intervention arm, the dietitian’s self-assessed stage of change for each participant to make dietary modifications were: precontemplation (26%, *n* = 20), contemplation (10%, *n* = 8), preparation (42%, *n* = 32), action (18%, *n* = 14) and maintenance (4%, *n* = 3).

### 3.2. Dietary Intakes (Expressed as Mean Servings ± SE) between Study Arms

The dietary intakes as assessed by the SDQ-AMD between study arms at baseline and at 3 months post-intervention are shown in Table 2. Unadjusted baseline dietary intakes between intervention and control participants were not significantly different (*p* > 0.05) and specific *p*-values can be found in Supplementary File S3. At the 3-month post-intervention follow-up, adjusted mean intake of nuts was significantly higher in the intervention arm than the control arm (3.96 ± 0.51 vs. 2.71 ± 0.32, *p* = 0.04). Unadjusted mean intakes at 3 months post-intervention can be found in Supplementary File S4.

### 3.3. Dietary Intakes (Expressed as Mean Servings ± SE) within the RCT Arms

#### Immediately Post-Intervention

Within the intervention arm, there were significant improvements in dietary intake at the immediate post-intervention follow-up compared to baseline (Supplementary Table S5). This included significant improvements in daily water intake (5.36 ± 0.27 vs. 4.63 ± 0.26; *p* = 0.01) and weekly intakes of: fish/seafood (2.36 ± 0.18 vs. 1.79 ± 0.17; *p* = 0.006), dark green leafy vegetables (1.95 ± 0.27 vs. 1.01 ± 0.17; *p* = 0.001) and eggs (3.78 ± 0.29 vs. 3.32 ± 0.25; *p* = 0.049). Non-significant improvements in intakes of total vegetables, fruit, red and processed meats, legumes, nuts, specified sweets and processed/prepared foods, alcohol and olive oil were also observed.

#### Three Months Post-Intervention

The dietary intakes at the 3-month post-intervention follow-up are shown in Table 2 and comparisons of these are presented for both arms. Participants who underwent the intervention had significantly higher age- and sex-adjusted mean intakes of dark green leafy vegetables (1.71 ± 0.22 vs. 0.99 ± 0.17, *p* = 0.003) and legumes (1.12 ± 0.16 vs. 0.69 ± 0.10, *p* = 0.02) at the 3-month follow-up than baseline. Furthermore, intakes of specified sweets and processed/prepared foods were reduced significantly (6.54 ± 0.58 vs. 8.31 ± 0.76, *p* = 0.01) compared to baseline. The control arm also showed significantly reduced intakes of specified sweets and processed/prepared foods compared to baseline (6.84 ± 0.62 vs. 8.82 ± 0.82, *p* = 0.0002).

**Figure 2:**
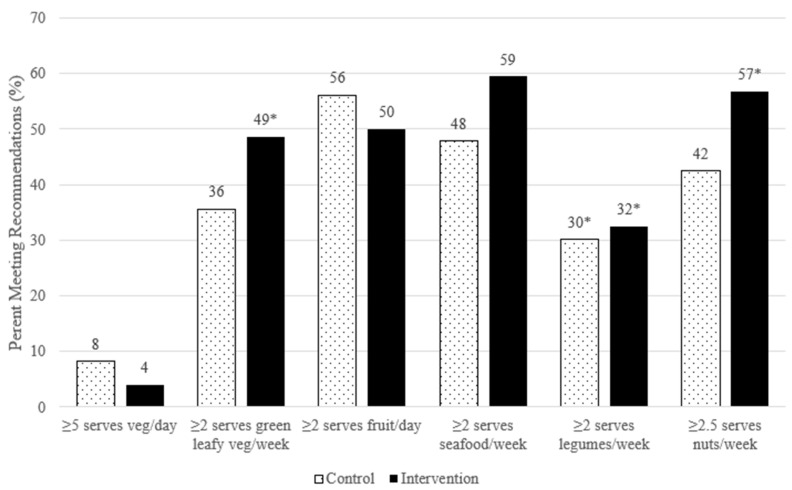
Proportion of participants meeting dietary goals at 3 months post-intervention. * Indicates a statistically significant (*p* < 0.05) increase within the study arm of the proportion of participants meeting the required intake compared to baseline. No significant difference in proportion was found between the study arms.

Figure 2 illustrates the proportion of participants at 3 months post-intervention from both arms meeting key dietary goals based on the Australian Dietary Guidelines [28] and recommendations presented in research literature for AMD [3,13,32,33,34,35]. There were statistically significant increases in the proportion of intervention participants meeting the goals for: dark green leafy vegetables (+18.9%, *p* = 0.01), legumes (+16.2%, *p* = 0.02) and nuts (+13.5%, *p* = 0.03) compared to baseline (Supplementary File S6). Within the control arm there was a significant increase in the proportion of participants meetings the recommendations for legumes (+14%, *p* = 0.01). No significant differences were found between the study arms at 3 months post-intervention.

### 3.4. Intervention Adherence and Acceptability

During the 4-month intervention period, an average of 6.5 (SD = 2.2) call attempts were made per intervention participant with 3.9 (SD = 0.6) successful calls. The total duration of calls per participant averaged 75.8 min (SD = 23.1 min).

Most participants (94.9%, *n* = 74) completed the feedback form, with almost all completers (98.6%, *n* = 73) being “satisfied” or “very satisfied” with both the evidence-based nutrition resource and telehealth calls; one participant was neutral to both. Similarly, 98.6% (*n* = 73) of completers would recommend the intervention to others, and all participants who completed the feedback form thought the intervention was worth participating in. Almost three quarters of the intervention participants (72%, *n* = 53) self-reported good to high adherence to the programme. Some participants cited reduced oral intake secondary to poor appetite or health, and inflexible diets due to established menus at nursing homes/aged care facilities as reasons for poorer adherence.

Below are some participants’ comments about the intervention programme. Participants are identified using individual identification numbers.

Most participants valued the monthly telephone calls, which were tailored according to each participant’s needs:

Simple explanations and good suggestions about food substitutes if different foods are not liked or tolerated.

(029)

Regular follow up which reinforced diet preference and benefits.

(033)

Talking to [the dietitian] when I was getting lazy cooking for myself nightly, encouraged me to go back to a healthy eating practice.

(142)

The phone calls review my previous month’s diet. It made me think about what I had eaten and encouraged me to eat better in the next month.

(146)

The evidence-based resource was also valuable to participants to help remind and prompt them about healthy eating practices:

I keep referring to the ‘Dietary Recommendations for AMD’ so that I’ll keep track.

(082)

It was more the reminder to keep on the healthy foods and what are the best to have.

(053)

I found the dietary sheets very helpful for planning the week meals and shopping.

(054)

A few participants reported having a healthy diet but appreciated the intervention to help reinforce their nutrition knowledge:

Confirmation of food choices. Recommendation of foods that were more beneficial.

(015)

Although I have a good diet it was useful to get guidance and keep up to date.

(108)

*Helpful advice...found it reassuring as my diet has always included the foods recommended so now more focused on eating them regularly*.

(130)

## 4. Discussion

This is the first study to evaluate an evidence-based telehealth intervention programme designed specifically to improve the dietary intakes and behaviours of people with AMD. Overall, the intervention was delivered as planned for most participants, with a total of 3.9 out of the intended four successful phone calls made per participant and an average total call duration of 75 min compared to the expected 80 min over the four months. The programme was also well received and appreciated by the participants and led to statistically significant and clinically meaningful improvements in dietary intakes over a 3-month follow-up period.

Noteworthy differences were observed between the baseline dietary intakes of key food groups among our study participants (with an average age in their late 70s), and the population-based intakes of Australians aged 71 years and older [36]. Our participants had a comparable vegetable intake to the 2.3 servings per day national average, however, both study arms consumed more than the 0.65 servings per week of leafy vegetables in the general older Australian population [36]. The participants’ mean intakes of fruit, nuts and legumes were also higher than the national average at 1.1 servings per day, 0.96 servings per week and 0.42 servings per week, respectively [36]. However, the average older Australian consumed more fish/seafood than our participants, at 2.0 servings per week [36]. Bread intake varied; participant consumption of wholemeal, grain, rye and sourdough was comparable or higher than the 4.1 servings per week average, and lower than the 6.1 servings per week average for white bread [36].

Some of the key dietary recommendations for AMD published in the literature are to include: at least two servings per week of fish/seafood, which is associated with reduced risk of early and neovascular AMD [3,4,5,13,37]; at least two servings per week of dark green leafy vegetables, which is recommended to obtain the benefits of dietary carotenoids [3]; and two to four eggs per week, which has been linked to reduced risk of AMD progression, with eggs additionally being a bioavailable source of lutein and zeaxanthin [5,38]. In our study, the intervention arm significantly increased their mean intakes to meet these particular recommendations immediately after the intervention. These improvements may be due to the programme’s emphasis on AMD-specific dietary advice rather than general healthy eating advice such as increasing total vegetable and fruit intake.

At the 3-month post-intervention follow up, intakes of dark green leafy vegetables continued to be significantly higher compared to baseline intakes, suggesting that this particular dietary modification may have been more feasible to incorporate and maintain in the diet than the other recommendations. Interestingly, total vegetable intake at 3 months post-intervention was not significantly different to baseline, at approximately two servings per day, despite a 73% increase in dark green leafy vegetable intake. This increase of an average of 0.72 servings per week of dark green leafy vegetables may have replaced participant intakes of other vegetable categories, as cooked green vegetables such as peas and broccoli decreased by 0.24 servings per week, thereby negating any meaningful changes in total vegetable intake post-intervention. Specific additional vegetable categories (e.g., root vegetables) were not captured in the SDQ-AMD to confirm this. Nonetheless, older adults are recommended to consume five servings of vegetables per day [28] and, therefore, consumption of this food group and its associated micronutrients continue to be inadequate among AMD patients. Additional tailored behaviour change strategies should be explored in future to increase total vegetable intake.

Mean fish/seafood intake at 3 months post-intervention continued to meet the recommended ≥ two servings per week [3] and was approximately 0.3 servings higher than baseline. This difference is equivalent to a 30 g increase over the week, where a serving of fish/seafood is 100 g according to the Australian Dietary Guidelines [28]. Although we did not find this result to be statistically significant, the research literature suggests that this increase has clinical importance for general health, as a multi-cohort cross-cultural study investigating the diets of older adults in Australia, Greece, Japan and Sweden reported that every 20 g increase in fish and shellfish intake was significantly associated with a 6% reduction in the risk of death, after accounting for ethnicity [39]. Comparatively, our control arm’s mean fish/seafood intake increased by approximately 10 g (0.1 servings) to 1.82 servings per week, which does not have statistical significance and is less likely to be of clinical significance. This suggests that more targeted interventions than standard brochures may be needed to change dietary behaviour.

The study by Darmadi-Blackberry et al. also reported that every 20 g increase in legume intake was significantly associated with an 8% reduction in the risk of death, irrespective of ethnicity [38]. In our study, both the intervention and control arms increased their mean legume intake by more than 20 g (where a serving of legumes is 150 g [28]) at the 3-month post-intervention follow-up compared to baseline. However, the increase within the intervention arm was 25% higher than the control arm, further supporting the effectiveness of the programme. As legumes are a source of low GI carbohydrates, the meaningful increase in the consumption of these foods, coupled with the reduction of high GI carbohydrates such as sweets and processed/prepared foods, white bread and alcohol, suggests that an overall improvement in dietary GI may have been achieved within the intervention arm. Comparatively, dietary GI within the control arm may not have improved as much, as intakes of white bread and alcohol increased despite a greater reduction in sweets and processed/prepared food intake. Future analysis of 6-month post-intervention data, including responses to the 145-item food frequency questionnaire, will confirm changes to dietary GI and glycaemic load.

In addition to improvements within the intervention arm, the programme appears to have led to a difference in dietary intakes between study arms at 3 months post-intervention. The most notable difference was a significantly higher mean intake of nuts in the intervention arm (four servings per week) compared to the control arm (2.7 servings per week). The intervention arm also achieved better intakes of other food items compared to the control arm, however, these were non-significant. Interestingly, mean intakes of total vegetables and fruit were non-significantly higher in the control arm than the intervention arm at this follow-up. Possible reasons for this difference could again be due to the programme’s focus on AMD-specific recommendations rather than the general healthy eating advice that was provided to the control arm or might be due to individual preferences for seasonal produce.

Overall, the positive dietary changes reported in this study may be the result of a culmination of factors. A study evaluating nutrition interventions amongst older adults reported that dietary modification was more successful in studies that included participants with a specific health condition [40]. This may be due to increased motivation to manage the condition, as most (60%) of our intervention participants were in the “preparation” or “action” stages of change at the start of the programme. Furthermore, collaborative goal setting and regular contact with a health professional, which are incorporated within the four As approach of our intervention [29], have been shown to be successful nutrition education intervention components, leading to better behavioral outcomes in older adults [29,40,41]. However, more significant dietary improvements may have been observed if our programme limited its focus to one or two messages [40], such as increasing intakes of dark green leafy vegetables and fish/seafood rather than also including messages around other food groups/items like nuts and legumes.

### Strengths and Limitations

There are several strengths to this study. Firstly, this is a novel programme designed to specifically improve the dietary intakes and behaviours of people with AMD and was tested using the “gold standard” RCT study design. Secondly, this programme involved a collaborative effort between accredited practising dietitians and experts in the field of AMD (retinal specialists and epidemiologists) to provide evidence-based care. Thirdly, this programme incorporates a telehealth component which is particularly suited to our study participants, who were typically older adults with functional limitations (vision and mobility) and other pre-existing health problems. Acceptance of a telehealth programme is especially relevant in light of the current coronavirus (COVID-19) pandemic, which has emphasised the importance of telehealth services to provide safe and accessible healthcare to vulnerable subsets of the population [42]. Our study findings therefore reinforce the advantages of telehealth programmes and confirm that safe and effective dietary advice and counselling can be provided to older adults in this format.

However, no study is without limitations. We primarily recruited participants from eye clinics where patients come for treatment and/or specialist review and, as a result, there was a higher proportion of late AMD cases enrolled in the RCT. Therefore, the results reported in this study may not reflect a typical population of AMD patients, as early AMD is the more prevalent form globally [1]. Furthermore, late AMD is also strongly associated with older age [1] and this is reflected by the mean age of our participants (intervention: 78.1 ± 8.1 vs. control: 77.9 ± 8.5). Consequently, we cannot discount that the more severe vision loss and older age associated with late-stage AMD could have limited the participants’ ability and/or willingness to make dietary modifications compared to those with early/intermediate AMD (who might also be comparatively younger in age). Moreover, our participants may have already made substantial dietary changes at an earlier stage or when they were first diagnosed with AMD, which could further limit any subsequent behaviour changes. However, it is important to note that our participants may have been more motivated than average patients with late AMD with potentially better diets, as they volunteered to participate in this study and the majority (60%) were preparing to or already making changes to their diet at the start of the intervention. Lastly, we acknowledge that usual dietary intake may not have been captured, as the SDQ-AMD tool collects actual intake in the last week of a limited number of food items. In addition, this tool does not include a question about supplement use and, therefore, we are unable to report on participants’ use of the recommended Age-Related Eye Disease Study (AREDS) supplements for AMD. To overcome these limitations, the food frequency questionnaire, which collects usual dietary intake of 145 items and supplement use in the last 12 months, is currently being re-administered alongside the SDQ-AMD at the final follow-up (i.e., 6 months post-intervention). These data will then be compared with baseline data to provide further valuable insight into the usual dietary intakes and behaviour changes of patients with AMD.

## 5. Conclusions

This telehealth programme was appreciated by the participants and has potential to provide the needed support to people with AMD to effectively improve their nutrition knowledge and dietary behaviours. Post-intervention, a significant difference in the intake of nuts was observed between study arms, with significant improvements within the intervention arm for intakes of dark green leafy vegetables, legumes and sweets and processed/prepared foods. Further analysis of 6-month post-intervention data will provide additional insight into the effectiveness of the programme.

## Figures and Tables

**Table 1:** Baseline characteristics of participants.

Baseline Characteristics	Intervention (*n* = 77)	Control (*n* = 78)	*p*-Value
Age (years)	78.1 ± 8.1	77.9 ± 8.5	0.88
Sex (% female)	50.7	64.1	0.09
Weight (kg)	75.0 ± 15.6	70.7 ± 14.0	0.08
Height (cm)	167.2 ± 11.5	161.5 ± 9.5	**0.002**
BMI (kg/m^2^)	26.8 ± 4.9	27.0 ± 5.4	0.82
Type of age-related macular degeneration (AMD):			
No. eyes with early AMD	6	0	
No. eyes with any late AMD	94	104	**0.01**
Cardiovascular disease (*n*, %)	29.0 (37.7)	26.0 (33.3)	0.57
Stroke (*n*, %)	8.0 (10.4)	7.0 (9.0)	0.77
High blood pressure (*n*, %)	49.0 (63.6)	49.0 (62.8)	0.92
High cholesterol (*n*, %)	41.0 (53.3)	38.0 (50.7)	0.75
Diabetes (*n*, %)	15.0 (19.7)	21.0 (26.9)	0.29
Kidney disease (*n*, %)	6.0 (7.8)	4.0 (5.2)	0.51
Physical activity (h/week)	3.6 ± 4.0	4.4 ± 7.7	0.41
No. smokers (%)	34 (44.7)	28 (35.9)	0.35

Bolded values indicate statistical significance, *p*-value < 0.05.

**Table 2:** Age- and sex-adjusted mean dietary intakes at baseline and 3 months post-intervention.

	Intervention (*n* = 74)			Control (*n* = 73)			Mean Difference (Intervention–Control) at 3 Months Post-Intervention	
	Baseline Mean Servings ± SE*	3 Months Post-Intervention Mean Servings ± SE	*p*-Value	Baseline Mean Servings ± SE	3 Months Post-Intervention Mean Servings ± SE	*p*-Value	Mean Difference ± SE	*p*-Value
Intake reported (per day):								
Total vegetables	2.15 ± 0.16	2.01 ± 0.17	0.47	2.12 ± 0.13	2.28 ± 0.21	0.43	−0.28 ± 0.27	0.31
Fruit	1.88 ± 0.13	1.84 ± 0.14	0.73	1.72 ± 0.12	1.85 ± 0.15	0.23	−0.02 ± 0.20	0.93
Water	4.64 ± 0.26	4.73 ± 0.34	0.71	4.76 ± 0.27	4.47 ± 0.50	0.51	0.26 ± 0.67	0.67
Intake reported (per week):								
Dark green leafy vegetables	0.99 ± 0.17	1.71 ± 0.22	**0.003**	1.16 ± 0.25	1.32 ± 0.20	0.53	0.39 ± 0.29	0.19
Cooked green vegetables	3.92 ± 0.50	3.68 ± 0.30	0.65	4.16 ± 0.51	3.53 ± 0.29	0.23	0.15 ± 0.42	0.72
Red meat	2.06 ± 0.16	2.37 ± 0.20	0.11	2.29 ± 0.20	2.44 ± 0.26	0.58	−0.07 ± 0.33	0.82
Processed meat	1.41 ± 0.20	1.39 ± 0.20	0.97	1.14 ± 0.16	1.35 ± 0.15	0.24	0.05 ± 0.25	0.86
Fish/seafood	1.75 ± 0.17	2.02 ± 0.18	0.17	1.73 ± 0.16	1.82 ± 0.22	0.68	0.20 ± 0.29	0.49
Legumes	0.69 ± 0.10	1.12 ± 0.16	**0.02**	0.84 ± 0.14	1.16 ± 0.17	0.08	−0.04 ± 0.24	0.86
Nuts	3.29 ± 0.5	3.96 ± 0.51	0.15	3.27 ± 0.39	2.71 ± 0.32	0.06	1.25 ± 0.60	**0.04**
Eggs	3.34 ± 0.25	2.92 ± 0.24	0.14	2.72 ± 0.24	2.47 ± 0.25	0.25	0.46 ± 0.34	0.18
Bread:								
Wholemeal, grain, rye, sourdough	5.06 ± 0.50	4.71 ± 0.48	0.58	4.53 ± 0.53	4.26 ± 0.63	0.49	0.46 ± 0.63	0.47
White	1.41 ± 0.30	1.33 ± 0.30	0.85	1.63 ± 0.31	1.73 ± 0.31	0.72	−0.41 ± 0.43	0.35
Cakes, biscuits, ice cream, processed potato, takeaway, sugar-sweetened beverages	8.31 ± 0.76	6.54 ± 0.58	**0.01**	8.82 ± 0.82	6.84 ± 0.62	**0.0003**	−0.30 ± 0.85	0.73
Alcohol	3.72 ± 0.83	2.93 ± 0.53	0.30	2.18 ± 0.48	2.58 ± 0.51	0.40	0.35 ± 0.74	0.64
Fats and oils:								
Olive oil	2.14 ± 0.28	2.05 ± 0.28	0.71	2.58 ± 0.30	2.59 ± 0.31	0.98	−0.54 ± 0.42	0.20
Other	5.72 ± 0.44	5.48 ± 0.39	0.59	6.57 ± 0.45	6.31 ± 0.50	0.57	−0.83 ± 0.63	0.19

* SE = standard error. Bolded values indicate statistical significance, *p*-value < 0.05.

## Supplementary Materials

The following are available online at https://www.mdpi.com/2072-6643/12/10/3083/s1, Figure S1: Proportion of participants meeting dietary goals at baseline, Table S1: CONSORT checklist, Table S2: The TIDieR Checklist, Table S3: Unadjusted mean dietary intakes at baseline, Table S4: Age-sex adjusted mean dietary intakes of the intervention arm at baseline and immediate post-intervention follow up, Table S5: Unadjusted mean dietary intakes at baseline and 3-months post-intervention.
