# Supplementary material for: Telephone-Delivered Dietary Intervention in Patients with Age-Related Macular Degeneration: 3-Month Post-Intervention Findings of a Randomised Controlled Trial
Source: Nutrients. 2020 Oct 10;12(10):3083. doi: 10.3390/nu12103083 (PMC7650817; doi:10.3390/nu12103083)
Supplement: Supplementary file 1 [file nutrients-12-03083-s001.zip › Additional File 3.docx]

Additional Table 3. Unadjusted mean dietary intakes at baseline

| Baseline | | | |
| --- | --- | --- | --- |
|  | Intervention  N = 77  Mean serves ± SD | Control  N = 78  Mean serves ± SD | P-value |
| Intake ‘*per day’*: |  |  |  |
| Total vegetables | 2.1 ± 1.4 | 2.1 ± 1.1 | 0.93 |
| Fruit | 1.9 ± 1.1 | 1.8 ± 1.1 | 0.66 |
| Water | 4.5 ± 2.3 | 4.7 ± 2.4 | 0.56 |
| Intake ‘*per week’*: | | | |
| Dark green leafy vegetables | 1.0 ± 1.5 | 1.3 ± 2.2 | 0.38 |
| Red meat | 2.1 ± 1.4 | 2.3 ± 1.8 | 0.43 |
| Processed meat | 1.4 ± 1.8 | 1.1 ± 1.4 | 0.34 |
| Fish/seafood | 1.8 ± 1.5 | 1.9 ± 1.9 | 0.76 |
| Legumes | 0.7 ± 0.9 | 0.9 ± 1.3 | 0.34 |
| Nuts | 3.3 ± 4.1 | 3.2 ± 3.5 | 0.87 |
| Eggs | 3.2 ± 2.2 | 2.8 ± 2.1 | 0.15 |
| Bread:  Wholemeal, grain, rye, sourdough  White | 4.9 ± 4.6  1.5 ± 3.1 | 4.5 ± 4.4  1.5 ± 2.6 | 0.60  0.97 |
| Cakes, biscuits, ice cream, processed potato, takeaway, sugar sweetened beverages | 8.3 ± 7.1 | 8.6 ± 6.9 | 0.80 |
| Alcohol | 3.6 ± 7.2 | 2.1 ± 4.0 | 0.12 |
| Fats and oils:  Olive oil  Other | 2.2 ± 2.5  5.8 ± 3.8 | 2.6 ± 2.7  6.4 ± 3.9 | 0.26  0.32 |
