# Supplementary material for: Telephone-Delivered Dietary Intervention in Patients with Age-Related Macular Degeneration: 3-Month Post-Intervention Findings of a Randomised Controlled Trial
Source: Nutrients. 2020 Oct 10;12(10):3083. doi: 10.3390/nu12103083 (PMC7650817; doi:10.3390/nu12103083)
Supplement: Supplementary file 1 [file nutrients-12-03083-s001.zip › Additional File 5.docx]

Additional Table 4. Age-sex adjusted mean dietary intakes of the intervention arm at baseline and immediate post-intervention follow up

| Intervention (n = 75) | | | |
| --- | --- | --- | --- |
|  | Baseline  Mean serves ± SE | Immediate Post-Intervention  Mean serves ± SE | P-value |
| Intake ‘*per day’*: |  |  |  |
| Total vegetables | 2.14 ± 0.15 | 2.45 ± 0.15 | 0.10 |
| Fruit | 1.86 ± 0.13 | 1.87 ± 0.11 | 0.90 |
| Water | 4.63 ± 0.26 | 5.36 ± 0.27 | **0.01** |
| Intake ‘*per week’*: | | | |
| Dark green leafy vegetables | 1.01 ± 0.17 | 1.95 ± 0.27 | **0.001** |
| Red meat | 2.07 ± 0.16 | 1.96 ± 0.16 | 0.51 |
| Processed meat | 1.38 ± 0.21 | 1.22 ± 0.18 | 0.41 |
| Fish/seafood | 1.79 ± 0.17 | 2.36 ± 0.18 | **0.006** |
| Legumes | 0.71 ± 0.10 | 0.95 ± 0.14 | 0.12 |
| Nuts | 3.26 ± 0.46 | 3.51 ± 0.30 | 0.58 |
| Eggs | 3.32 ± 0.25 | 3.78 ± 0.29 | **0.049** |
| Bread:  Wholemeal, grain, rye, sourdough  White | 4.99 ± 0.52  1.40 ± 0.35 | 4.95 ± 0.50  1.41 ± 0.39 | 0.93  1.00 |
| Cakes, biscuits, ice cream, processed potato, takeaway, sugar sweetened beverages | 8.22 ± 0.75 | 6.88 ± 0.71 | 0.09 |
| Alcohol | 3.74 ± 0.82 | 2.96 ± 0.55 | 0.25 |
| Fats and oils:  Olive oil  Other | 2.17 ± 0.28  5.68 ± 0.44 | 2.18 ± 0.28  5.63 ± 0.36 | 0.97  0.90 |
