# Supplementary material for: Telephone-Delivered Dietary Intervention in Patients with Age-Related Macular Degeneration: 3-Month Post-Intervention Findings of a Randomised Controlled Trial
Source: Nutrients. 2020 Oct 10;12(10):3083. doi: 10.3390/nu12103083 (PMC7650817; doi:10.3390/nu12103083)
Supplement: Supplementary file 1 [file nutrients-12-03083-s001.zip › Additional File 4.docx]

Additional Table 5. Unadjusted mean dietary intakes at baseline and 3-months post-intervention

|  | Intervention (n = 74) | | | Control (n = 73) | | | Mean difference (intervention – control) at 3-months post-intervention | |
| --- | --- | --- | --- | --- | --- | --- | --- | --- |
|  | Baseline  Mean serves ± SD | 3-months post-intervention  Mean serves ± SD | P-value | Baseline  Mean serves ± SD | 3-months post- intervention  Mean serves ± SD | P-value | Mean difference ± SD | P-value |
| Intake reported  ‘*per day’*: |  |  |  |  |  |  |  |  |
| Total vegetables | 2.2 ± 1.4 | 2.0 ± 1.5 | 0.44 | 2.1 ± 1.1 | 2.3 ± 1.8 | 0.43 | -0.3 ± 1.7 | 0.29 |
| Fruit | 1.9 ± 1.1 | 1.8 ± 1.2 | 0.74 | 1.7 ± 1.0 | 1.9 ± 1.3 | 0.22 | -0.0 ± 1.2 | 0.89 |
| Water | 4.6 ± 2.3 | 4.8 ± 3.0 | 0.68 | 4.7 ± 2.5 | 4.4 ± 4.3 | 0.58 | 0.3 ± 3.7 | 0.60 |
| Intake reported  ‘*per week’*: |  |  |  |  |  |  |  |  |
| Dark green leafy vegetables | 1.0 ± 1.5 | 1.7 ± 1.9 | **0.0004** | 1.2 ± 2.1 | 1.3 ± 1.7 | 0.52 | 0.4 ± 1.8 | 0.23 |
| Red meat | 2.1 ± 1.4 | 2.4± 1.7 | 0.10 | 2.3 ± 1.8 | 2.4 ± 2.3 | 0.65 | -0.0 ± 2.0 | 0.92 |
| Processed meat | 1.4 ± 1.9 | 1.4 ± 1.7 | 0.97 | 1.1 ± 1.4 | 1.3 ± 1.3 | 0.26 | 0.1 ± 1.5 | 0.74 |
| Fish/seafood | 1.8 ± 1.6 | 2.0 ± 1.6 | 0.21 | 1.7 ± 1.3 | 1.8 ± 2.0 | 0.66 | 0.2 ± 1.8 | 0.51 |
| Legumes | 0.7 ± 0.9 | 1.1 ± 1.4 | **0.02** | 0.8 ± 1.3 | 1.2 ± 1.5 | 0.09 | -0.0 ± 1.4 | 0.89 |
| Nuts | 3.3 ± 4.0 | 4.0 ± 4.4 | 0.15 | 3.2 ± 3.5 | 2.7 ± 2.7 | 0.11 | 1.3 ± 3.7 | **0.03** |
| Eggs | 3.2 ± 2.2 | 2.9 ± 2.1 | 0.16 | 2.7 ± 2.1 | 2.5 ± 2.1 | 0.27 | 0.5 ± 2.1 | 0.17 |
| Bread:  Wholemeal, grain, rye, sourdough  White | 5.1 ± 4.6  1.4 ± 3.0 | 4.8 ± 4.2  1.3 ± 2.5 | 0.60  0.87 | 4.5 ± 4.5  1.6 ± 2.6 | 4.2 ± 3.6  1.7 ± 2.7 | 0.51  0.73 | 0.6 ± 3.9  -0.4 ± 2.6 | 0.38  0.37 |
| Cakes, biscuits, ice cream, processed potato, takeaway, sugar sweetened beverages | 8.2 ± 7.2 | 6.6 ± 5.3 | **0.01** | 8.9 ± 7.0 | 6.8 ± 5.3 | **0.0002** | -0.2 ± 5.3 | 0.83 |
| Alcohol | 3.6 ± 7.3 | 3.0 ± 4.6 | 0.36 | 2.2 ± 4.1 | 2.6 ± 4.4 | 0.44 | 0.4 ± 4.5 | 0.60 |
| Fats and oils:  Olive oil  Other | 2.1 ± 2.5  5.8 ± 3.9 | 2.1 ± 2.5  5.5 ± 3.4 | 0.74  0.58 | 2.6 ± 2.6  6.6 ± 3.9 | 2.6 ± 2.7  6.3 ± 4.3 | 0.91  0.54 | -0.5 ± 2.6  -0.8 ± 3.9 | 0.21  0.20 |
